# Bone Marrow Transplantation as Therapy for Ataxia-Telangiectasia: A Systematic Review

**DOI:** 10.3390/cancers12113207

**Published:** 2020-10-31

**Authors:** Bruna Sabino Pinho de Oliveira, Sabrina Putti, Fabio Naro, Manuela Pellegrini

**Affiliations:** 1 Department of Anatomical, Histological, Forensic Medicine and Orthopedic Sciences, Faculty of Pharmacy and Medicine, Sapienza University, Via Scarpa 16, 00161 Rome, Italy; bruna.sabino@uniroma1.it (B.S.P.d.O.); fabio.naro@uniroma1.it (F.N.); 2 Institute of Biochemistry and Cell Biology, IBBC-CNR, Campus Adriano Buzzati Traverso, Via Ramarini 32, 00015 Monterotondo, Rome, Italy; sabrina.putti@cnr.it

**Keywords:** ATM, A-T patients, A-T treatments, bone marrow transplantation

## Abstract

**Simple Summary:**

Ataxia-Telangiectasia is a rare neurodegenerative disease and patients die in their early forties mainly because of immunodeficiency, leukemia and lymphoma. In this work we describe the different routes of diagnosis, the recent treatments, and the emerging therapies for the disease. Bone marrow transplantation from siblings or an unrelated donor is becoming an option of therapy for selected Ataxia-Telangiectasia patients to deal with the immunodeficiency and to prevent leukemia and lymphoma. The patients require a non-myeloablative conditioning before bone marrow transplantation because they are radiosensitive to ionizing radiations and radiomimetics. The eligibility of patients for transplantation such as the range of age, spectrum of clinical features, and conditioning are still to be determined, transplant protocol guidelines must be defined and longer follow-ups are necessary to evaluate the risks and side effects associated with transplantation.

**Abstract:**

Ataxia-Telangiectasia (A-T) is a rare autosomal recessive disorder, first reported in 1926, caused by a deficiency of ATM (Ataxia-Telangiectasia Mutated) protein. The disease is characterized by progressive cerebellar neurodegeneration, immunodeficiency, leukemia, and lymphoma cancer predisposition. Immunoglobulin replacement, antioxidants, neuroprotective factors, growth, and anti-inflammatory hormones are commonly used for A-T treatment, but, to date, there is no known cure. Bone marrow transplantation (BMT) is a successful therapy for several forms of diseases and it is a valid approach for tumors, hemoglobinopathies, autoimmune diseases, inherited disorders of metabolism, and other pathologies. Some case reports of A-T patients have shown that BMT is becoming a good option, as a correct engraftment of healthy cells can restore some aspects of immunologic capacity. However, due to a high risk of mortality as a result of a clinical and cellular hypersensitivity to ionizing radiation and radiomimetic drugs, a specific non-myeloablative conditioning is required before BMT. Although BMT might be considered as one promising therapy for the treatment of immunological defects and cancer prevention in selected A-T patients, the therapy is currently not recommended or recognized and the eligibility of A-T patients for BMT is a point to deepen and deliberate.

## 1. Introduction

Ataxia-Telangiectasia (A-T) is a rare autosomal recessive disorder, characterized by a progressive cerebellar neurodegeneration, immunodeficiency, infertility, and cancer predisposition, with high incidence of leukemia and lymphoma [1,2]. A-T is caused by a deficiency of ATM (Ataxia-Telangiectasia Mutated) protein, encoded by a gene at chromosome 11q23 in humans [3]. The ATM protein belongs to the family of phosphatidylinositol 3-kinases that share homologies in the catalytic domain. The kinase functions as a guardian of genome integrity acting on cell-cycle checkpoints, apoptosis, gene expression, and DNA repair after DNA double-strand breaks (DSBs) [4]. DSBs are generated in the cells by exogenous and endogenous insults such as ionizing radiation, chemical reagents, replication, DNA demethylation, meiotic recombination, and during the generation of antibody diversity in the immune system.

ATM is recruited and activated by the MRN protein complex at DSBs. A phosphorylation- acetylation cascade sustains ATM activation by c-Abl tyrosine kinase and chromatin-bound TIP60 acetyltransferase [5]. Following activation, ATM acts as a DSB transducer activating a plethora of downstream effectors and orchestrating the DNA damage response [4].

Due to their critical role in genome stability, ATM mutations cause several body system malfunctions [6]. Among them, immunodeficiency is found in two-thirds of A-T patients affecting both humoral and cellular immunity [7,8], because of the V(D)J recombination impairment that affects B and T cell maturation [9]. Bone marrow transplantation (BMT) is a potential therapeutic approach for A-T patients, as reconstitution of cells with functional ATM can rescue some aspects of immunodeficiency [1,7,10].

In this review, we provide a perspective of A-T syndrome in the classical and mild forms, its diagnosis, the current treatments, and therapies to ameliorate the symptoms. We then discuss the relevancy of novel gene therapy strategies developed in cell lines and mouse models of A-T. We end by describing the therapeutic approach of BMT in Atm-deficient mice and summarizing case reports of A-T children recently subjected to BMT from siblings or matched unrelated donors.

## 2. Ataxia-Telangiectasia Syndrome: Diagnosis

Ataxia-telangiectasia (A-T) is a complex disorder clinically represented by a wide variability in patients [6,11]. Affected individuals can show early childhood-onset progressive cerebellar decline (ataxia), telangiectasia, predisposition to progressive bronchopulmonary disease, and cancer development, especially of lymphoid origin. A-T patients are also characterized by immunodeficiency with hypoplasia or agenesis of the thymus gland, clinical and cellular hypersensitivity to ionizing radiation and radiomimetic drugs. Usually, they show premature aging with progeria-type hair and skin changes, growth retardation, endocrine abnormalities, gonadal atrophy, choreoathetosis with dystonic posturing of hands and feet, osteoporosis, insulin-resistant diabetes. Mental retardation is not commonly seen in A-T patients [2,6,12].

A-T patients exhibit different phenotypes accordingly with the type of mutation carried by the ATM gene and with levels of ATM protein and kinase activity [6,13,14]. The majority of A-T patients have ATM truncating mutations, which encode highly unstable protein fragments that are not detected by Western blot analysis and that have lost kinase activity, leading to total loss of function of the ATM protein [2,6,14]. These patients exhibit the classical phenotype of A-T syndrome. The classic clinical presentation can also be observed in patients with residual ATM protein detectable by Western blot but lacking in kinase activity [6]. A variant form of A-T referred to as a milder phenotype, results from either leaky splice site mutations or missense mutations allowing the production of mutant ATM protein with kinase activity [13,15]. In this variant, there is a correlation between the milder presentation and progression of the syndrome and the degree of ATM kinase activity [6,14]. Patients with the milder variant of A-T commonly present later onset or slower neurological progression and a lower risk of systemic complications, except for an increased risk of malignancy [13,14,15]. Siblings and parents of A-T patients carrying heterozygous ATM mutation can present increased health risks and decreased life expectancy due to ischemic heart disease and increased susceptibility to develop cancer, especially breast and digestive tract cancers [15,16].

The A-T diagnosis is made by association of clinical features and specific laboratory findings, such as cerebellar atrophy on magnetic resonance imaging and lack of voluntary movement coordination, including gait abnormality, elevated serum alpha-fetoprotein (AFP) levels after two years of age, low serum levels of immunoglobulins (IgA, IgG, IgG subclasses and IgE), and lymphopenia [2,6,11]. The diagnosis confirmation is done by the assessment of absence or deficiency in the ATM protein and/or ATM kinase activity in cultured cell lines from lymphocytes or skin biopsies and the detection of mutations in the ATM gene [6].

## 3. Ataxia-Telangiectasia Syndrome: Current Treatments and New Emerging Therapies

Since there are no cures for A-T yet, the management and treatment for this syndrome are symptomatic and supportive, aiming to give patients a more comfortable life and to alleviate the symptoms [6,17]. As A-T is a multisystem disease, a “multidisciplinary therapy” is required to interfere with the progressive neurodegeneration, correct the immunodeficiency, diminish the proneness to cancer, and alleviate bronchial complications [17,18]. Immunoglobulin replacement therapy is recommended only to patients with life-threatening or frequently recurring infections [12]. For the other patients, it is recommended the use of inactivated vaccines as normal childhood vaccination routine [6,18]. A-T patients’ cells exhibit reduced levels of antioxidant capacity [19,20], and oxidative stress can promote macromolecular damage in affected subjects [6,17]. Therapies with antioxidants are also being exploited both in ATM-deficient cells [21,22] and in Atm-deficient mice [23]. Since the generation of excessive free radicals creates life-threatening DNA lesions, antioxidant therapies are recommended for A-T patients, although there are no published clinical trials showing therapeutic advantages [11,24]. One study showed that superoxide dismutase (SOD) mimetics, may also confer radioprotection for A-T patients, as they were able to significantly decrease the levels of reactive oxygen species (ROS) both in the presence and in the absence of irradiation (IR), and they were able to reduce both IR-induced cell death and apoptosis in A-T cells [25]. It has also been suggested that a wide-spectrum of neuroprotective factors such as insulin-like growth factor 1 (IGF-1) could be beneficial in cerebellar ataxia treatments [26], and a clinical trial is being performed at the “Johann Wolfgang Goethe University Hospital”, Frankfurt, Germany (NCT01052623). A clinical trial showed that the administration of growth hormone in A-T patients increased the height of patients, but did not affect lymphocytes and ataxia subsets [27]. Glucocorticoids are powerful anti- inflammatory hormones, capable of crossing the blood−brain barrier, and for this reason they are used to treat the neurological symptoms of A-T as reported from case reports [28,29]. Positive effects of a 6-month treatment with erythrocyte-delivered dexamethasone (EryDex System, EryDel SPA, Italy) were reported with none of the side effects commonly associated with steroid treatments [30,31]. After 24 months of treatment the patients experienced a continuous neurological improvement [32]. Menotta et al., 2012 [33] suggested that dexamethasone is able to promote a noncanonical splicing event in the ATM gene, that allows the cell a second chance to produce the ATM protein, even if with reduced functions. This event could in part explain the effects of dexamethasone treatment on the neurological deterioration of A-T patients. Betamethasone therapy demonstrated short-term improvements of neurological symptoms for ataxia, however, a better understanding of the action mechanism is needed for consideration as a new approach for this disease [28,29,34]. Indeed, to date, the specific role of steroids has not been established and the clinical trials have not yet conclusively shown benefit. The generation of a rat *Atm^−/−^* model offered the opportunity to better study the neurological phenotype of the syndrome [35,36], and the study was able to show that low dose betamethasone treatment can break the neuroinflammatory loop in these *Atm^−/−^* rats [35].

Di Siena et al., 2018 [37] demonstrated that the reactivation of ATM reversed the A-T phenotype in *Atm^−/−^* mice, indicating that gene therapy can be a hopeful strategy for A-T patients. Since the majority of ATM mutations cause premature protein truncation, mutation-target therapies attempt to restore ATM gene function by read-through of premature termination codons by using aminoglycosides or small molecule read-through (SMRT) compounds and correction of ATM splicing mutations with antisense morpholino oligonucleotides are gaining attention [2,17,38,39,40]. A clinical trial using antisense oligonucleotides (ASO) drugs is recently under investigation by the Dr. Timothy Yu Group at Boston Children’s Hospital (www.theyulab.org). A patient-specific drug is being engineered to bind to the mRNA and help the cell to correctly splice the exons, making the cell able to produce a certain amount of functional ATM. This personalized medicine seems promising, especially for the youngest patients who still do not show neurodegeneration. Indeed, these drugs pass the brain−blood barrier and might be able to produce ATM in brain cells. Unfortunately, this is a high-cost treatment because it is very patient-specific and limited to certain splicing aberrations. Mutation-target therapy is another valuable approach to several genetic diseases, including A-T [11]. CRISPR/Cas9-assisted gene correction combined with piggyBac transposon system was recently proven to efficiently correct ATM mutations in iPS cells (induced pluripotent stem cells) collected from two A-T patients [41]. Alternatively, attempts to insert a functional ATM gene through viral vectors are also a promising line of investigation, despite the low infection efficiency and the low viral titers due to ATM cDNA large size. Carranza et al., 2018 [42] was able to reconstitute the A-T phenotype in patient cells using lentiviral vectors containing a full-length ATM cDNA. Moreover, herpes simplex virus type 1 (HSV-1) alone or in association with adeno-associated virus (AAV) was also used as a vector to insert ATM cDNA in A-T human cells and correct pathological aspects of the cellular phenotype [43,44]. A low amount (~10%) of normal ATM kinase activity is sufficient to improve the A-T phenotype, suggesting that gene therapy approaches do not necessarily need to restore normal levels of ATM [14,17].

## 4. Bone Marrow/Hematopoietic Stem Cell Transplantation as a Therapeutic Approach

### 4.1. Bone Marrow Transplantation in the Mouse Model

Bone marrow transplantation is extensively used as treatment of many disorders involving bone marrow elements [45]. For A-T syndrome it was hypothesized that hematologic abnormalities could be overcome by replacing the hematopoietic compartment of Atm-deficient mice [1]. Since even a low-dose of irradiation therapy increased lethality in Atm-deficient mice [46], a non-myeloablative conditioning, consisting of a first dose of 0.5 mg anti-CD4 antibody and 1 mg anti-CD8 antibody 7 days before BMT and a second dose of each antibody, together with 200 mg/kg cyclophosphamide 1 day before BMT, was used to achieve engraftment of wild-type donor hematopoietic stem cells (HSCs) [1]. This type of conditioning was sufficient to induce full donor-type chimerism, and the BMT was able to restore immune function and to prevent the onset of thymic lymphoma in the Atm-deficient mice [1]. To further investigate the potential migration of bone marrow-derived cells (BMDCs) into the tissues of recipient animals, Pietzner et al., 2013 [10] transplanted green fluorescent protein (GFP)-expressing Ataxia-Telangiectasia mutated (Atm)-competent BMDCs into Atm-deficient mice. By this technique it was shown that donor BMDCs migrated into the bone marrow, blood, thymus, spleen, and lung tissue of Atm-deficient mice. BMT inhibited thymic lymphomas, normalized T-lymphocyte populations, prolonged lifespan, and significantly improved the phenotype of Atm-deficient mice. Similar results of BMT were observed by Duecker et al., 2019 [7]. Through the analysis of naïve CD4 and CD8 T-cells (CD62L^high^CD44^low^), the authors showed, after transplantation, the recovery of Atm-deficient mice, characterized by robust lymphopenia. Although BMDCs do not cross the brain−blood barrier, hematopoietic stem cell transplantation (HSCT) appears to be a feasible strategy to treat at least immunodeficiency and avoid T-cell to drive cancer in A-T patients [47,48].

### 4.2. Hematopoietic Stem Cell Transplantation in Human A-T Patients

**Figure 1:**
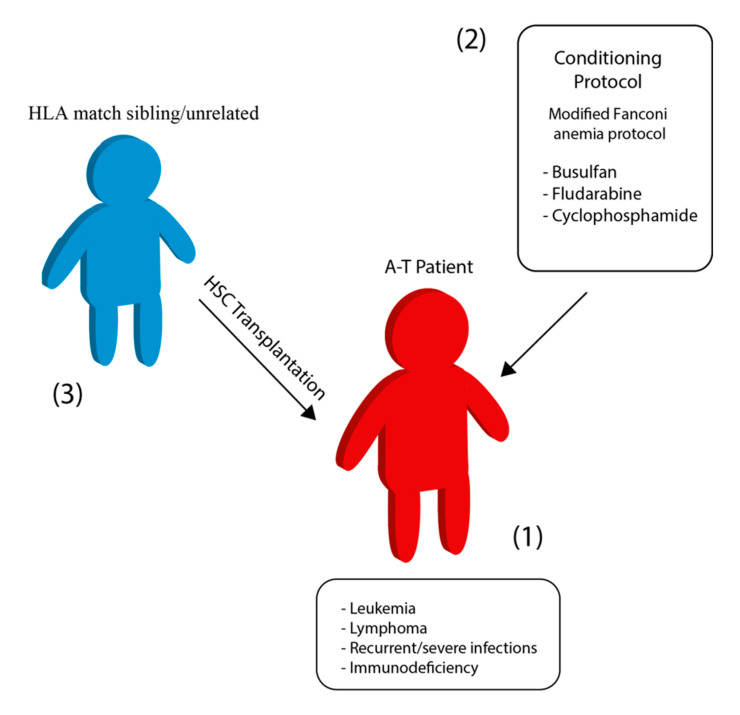
Hematopoietic stem cell transplantation in A-T patients. Young A-T patients with severe clinical manifestation (**1**) were conditioned with a modified Fanconi anemia protocol (**2**) for transplantation of hematopoietic stem cells (HSC) from their sibling or unrelated donor with perfectly matched human leukocyte antigen (HLA) (**3**).

In the early 1970s a boy with A-T and IgA deficiency was found with transient improvements in the immune system and normal IgA level several months after an infusion of bone marrow cells from a compatible healthy sibling [49]. However, no evidence of chimerism was detected in his peripheral blood lymphocytes and bone marrow cells. More recently, a Turkish child of 22 months of age, whose A-T diagnosis was made postmortem, was transplanted for a misdiagnosed immunodeficiency and died 8 months after, due to hepatic failure and encephalopathy [50]. Grounded in the data provided on mouse models, BMT was applied in human A-T patients (Figure 1, Table 1 and Table 2). In 2013 it was reported the first long-term survival of an A-T patient (PT1) after a peripheral blood stem cell transplantation at the age of 3 years old [51]. A modified protocol of the conditioning used in the Fanconi anemia disease (GEFA02; [52]) was employed to prevent graft rejection. The patient did not develop any symptoms of either acute or chronic graft-versus-host disease (GvHD), although presented mixed chimerism of 33% donor cells on day +15. He then received donor lymphocyte infusions at 4, 5, 8 and 10 months after transplantation. The follow-up, 3.5 years after the transplantation, stated that the patient was in complete hematological remission with normal blood count values. A more recent follow-up, published 8 years after transplantation [53], reported that the patient remained in leukemia remission, he was attending primary school but showed coordination deficits and when he was around age 12 he lost the ability to walk, becoming wheelchair-bound. Regarding the therapy, he did not require intravenous immunoglobulin substitution, he did not present any severe infection and the mixed chimerism percentage was stabilized in the range of 60% to 80% donor cells.

The next reported case of BMT in an A-T patient was published in 2016. A 13-year-old boy who also developed EBV-positive non-Hodgkin lymphoma was cured by chemotherapy associated with allogenic-matched sibling HSCT (allo-HSCT) [54]. The conditioning used was based on the same Fanconi anemia protocol (GEFA02), as the previous report, with association of fludarabine, busulfan and cyclophosphamide. Due to CD20-positive malignancy and an HLA (human leukocyte antigen)-identical sibling donor was available, the patient also received two doses of Rituximab for conditioning, and T-cell-depleting antibodies were not used. Donor chimerism was observed at day +32 and it was maintained to 100% of donor cells until his last follow-up at 1 year of age. Neutrophil engraftment was achieved on day +19 and acute or chronic GvHD were not observed. During the treatment, the patient developed severe mucosal toxicity, hemorrhagic cystitis and severe veno- occlusive disease. Mechanical ventilation became necessary after the patient developed an adult-type respiratory distress syndrome. Starting from 10 months, the percentage of CD19+, CD16+/CD56+, CD8+, CD3+, and CD4+ cells were increasingly normalized, and complete reconstitution of cells was observed 30 months after HSCT. Immunoglobulin reconstitution began at 6 months and after 30 months, IgM, IgG and IgA levels were normal, only IgG2 levels remained slightly reduced. As reported by Ussowicz et al., 2013 [51], this patient tolerated the therapy and achieved remission, but he did not tolerate the conditioning well, developing life-threatening toxicity in several organ systems.

More recently, it was published a long-term follow-up of 3 children with A-T after 112, 63 and 26 months since HSCT [53]. The first one being a new follow-up of the patient reported in 2013 [51] and previously cited in this review. The other 2 children (PT2, 12 months old and PT3, 23 months old) presented the classic version of A-T and based on previous experience with reported A-T patients, the conditioning protocol used was the modified GEFA03. They survived the procedure without significant adverse effects, showing mild mucosal toxicities and maintaining oral feeding ability throughout the transplant period. The patients were engrafted and lymphocyte reconstitution was observed. PT3 achieved stable CD4+ and CD8+ T cell counts after 6 months, although PT2 needed 24 months to achieve the same, most likely caused by a MabCampath serotherapy. PT2 required regular intravenous immunoglobulin supplementation beyond 6 months after transplantation, and IgG production recovered 3 years after HSCT. None of the patients developed pulmonary infections during the observation period, and only PT2 needed hospital admission for a catheter-related infection. Both children are in their first decade of life and exhibit mild neurologic deficits, PT2 head magnetic resonance revealed signs of cerebellar atrophy. No improvement was observed in growth and weight gain after HSCT, which remained poor, however, gastrostomy tube feeding was not considered by patients’ families.

Bakhtiar et al., 2018 [55] reported a 6-year follow-up of a pre-emptive allogenic HSCT in a 4-year-old boy with A-T. At the age of 3 he presented with upper respiratory infections and analysis revealed very low naïve T cells, absence of IgA and low IgG2 and IgG4. Clinical symptoms such as intermittent gait instability and frequent falls together with multiple telangiectasia on skin and conjunctiva suggested an A-T diagnosis that was confirmed by detection of a compound heterozygous mutation in the ATM gene causing a complete lack of the ATM protein. The patient also developed granulomas in the hands and elbow. The bone marrow from an HLA identical sibling was used to perform HSCT and a reduced intensity conditioning (RIC) regimen with fludarabine, cyclophosphamide and rabbit anti-thymocyte globulin was chosen. The engraftment was already observed by day +15, with an initial mixed chimerism in whole blood of 10–20% although CD3+ reached over 90% over time. The HSCT was able to correct T-cell lymphopenia by expansion of naïve and memory CD4+ T-cells, CD19+ and CD8+, moreover, there was an increase of IgA and IgG2 to normal levels. Analyses of ATM protein showed a complete renewal in peripheral blood cells. Clinically the patient gained height and weight, exhibited a milder progression of ataxic symptoms compared to age-matched A-T patients and granulomas were in complete remission during the 6 years of follow-up. Regarding the treatment in this patient, no signs of acute toxicity were observed and it provided complete immunological reconstitution along with the remission of granulomas without any need for immunosuppression or immunoglobulin replacement. The authors suggest that pre-emptive allogenic HSCT might be an early treatment of choice in some A-T patients at high risk of hematological malignancy [55].

## 5. Remarks

A-T is a very complex disorder and since its discovery many efforts have been focused on understanding the clinical features of the disease and its precise and early diagnosis, on investigating the molecular mechanisms altered in the pathological features and on finding possible therapies for A-T patients.

A clinical guidance for diagnosis and treatment of Ataxia Telangiectasia in children has been created (http://www.Atsociety.org.uk/data/files/William/A-T_Clinical_Guidance_Document_Final.pdf) and since 2016 the Global A-T Family Data Platform (https://www.atfamilies.org/) and the A-T International registry (http://www.atregistry.eu/) are available for patients, their parents, scientists, and physicians.

Recent studies on a very small and highly selected proportion of A-T patients, with clinical complications for which BMT transplant is already established, have shown that BMT and, in particular allo-HSCT, increase T lymphocytes and immunoglobulins and prevent severe infections or respiratory symptoms and signs of lung disease, in the short period of the follow-up. However, BMT was not able to evidently ameliorate the neurological symptoms and disability, which for most patients are the most important and quality-of-life limiting aspects of the disease, nor to rescue body growth and the serum AFP levels of A-T patients. A longer period of observation is necessary to understand the impact of HSCT on lymphoma and leukemia predisposition. To date, only a few A-T children have been transplanted with HSCs because of the high risk of mortality due to excessive toxicities and the ethical eligibility to perform HSCT on A-T patients remains controversial [53,54]. The patients with recurrent infections, severe manifestations of immune deficiency and children after cancer therapy are the most compelling candidates to benefit from this therapy, despite the difficulties to predict the outcome of single cases or small group treatment [53]. An early treatment performed during stages of limited disability could provide better results for this therapeutic option [7,55]. For all these reasons transplant guidelines are absolutely required for HSCT in A-T.

Since allo-HSCT in A-T is an invasive and risky treatment, transplant guidelines are absolutely required before considering pilot studies and randomized controlled trials.

Moreover, although allo-HSCT is a potentially curative treatment for several hematologic and immunological diseases, it is known that side effects including chronic GVHD, infectious and secondary malignancies occur in the long-term period post-transplantation [56].

For these reasons, it is fundamental to inform the patients and their parents of all possible deleterious late effects of BMT.

## 6. Conclusions

Primary immunodeficiency of Ataxia-Telangiectasia might ameliorate after bone marrow transplantation and consequently cancer prevention can be observed. Further studies with an increased cohort of patients and a longer period of follow-up are required to validate the efficacy of HSCT in A-T patients and importantly to monitor the associated risks of transplantation. Future designs of the bone marrow therapy should be considered in association with ATM correction/replacing techniques, making it possible to rescue an A-T patient’s own hematopoietic cells and to perform an autologous transplantation. This would limit the toxic response of the conditioning protocols and the heterologous transplantation. The development of gene therapy strategies will certainly increase our understanding of ATM functions and hopefully open the way for better treatments of A-T syndrome.

## Figures and Tables

**Table 1:** Bone marrow transplantation in A-T children.

Patient	Age	Clinical Symptoms	BMT (Year)	Donor	Conditioning Protocol (I.V.)	Follow-Up	Reference
1	22 months	Recurrent severe respiratory infections; hepatosplenomegaly; Hyper-IgM phenotype.	N/S	HLA-identical sibling	Treosulfan (3 × 12 g/m^2^) Fludarabine (5 × 30 mg/m^2^) ATG- Fresenius (3 × 20 mg/kg)	At the age of 30 months: increasing IgG levels, B cell count increased dramatically, and neutrophils declined; liver enzymes were elevated. Died at the age of 32 months, due to fulminant hepatic failure.	[50]
2 (PT1)	3 years	Leukemia; scheduled for high-risk chemotherapy with subsequent allo-HSCT	2009	HLA-matched sibling	Busilvex (0.5 mg/kg, 2 times/day; total dose 2 mg/kg) Fludarabine (30 mg/m^2^; total dose 150 mg/m^2^) ATG-Fresenius (20 mg/kg)	No symptoms of either acute or chronic GvHD; complete hematological remission; Did not worsen the patient’s neurological status. 8 years after transplantation: remains in leukemia remission; attends primary school; coordination deficits that make him wheelchair-bound; does not require intravenous immunoglobulin substitution and did not suffer from serious infections.	[51,53]
3	13 years	Cervical lymphadenopathy; prominent Waldeyer’s lymphatic ring; enlarged tracheal and intrapulmonary lymph nodes; cutaneous infiltration of the abdominal wall; infiltration of the right mastoid bones and the right middle ear; EBV-positive non-Hodgkin lymphoma	2014	HLA-identical sibling	Fludarabine 180 mg/m^2^, Busilvex (1.6 mg/kg) Cyclophosphamide (40 mg/kg) Rituximab (2 × 375 mg/m^2^)	Life-threatening toxicity in several organ systems due to conditioning; CD19+ and CD16+/CD56+ cells reconstituted 10 months post HSCT; total lymphocyte count and CD8+ cells normalized at 18 months; CD4+ cells at 30 months post HSCT. No acute or chronic GvHD was observed. Able to sit and stand without support; walks a few steps with assistance.	[54]
4 (PT2)	12 months	undefined, radiosensitive, severe combined immunodeficiency disease	N/S	HLA-10/10 matched unrelated	Busulfan (2 × 0.5 mg/kg/d) Fludarabine (6 × 30 mg/m^2^) Cyclophosphamide (2 × 20 mg/kg/d) MabCampath (1 × 0.25 mg/kg and 3 × 0.5 mg/kg/d)	Stage I acute skin graft-versus-host disease, which resolved completely with methylprednisolone 1 mg/kg/day after 3 days; Readmitted and treated with methylprednisolone for Coombs-positive hemolytic anemia; 8 months after SCT, polyclonal T cell repertoire was observed. Neurologic symptoms were observed in the second year of life, and A-T diagnosis was made 2 years after SCT.	[53]
5 (PT3)	23 months	Preemptive transplantation was performed	N/S	matched unrelated	Busulfan (2 × 0.5 mg/kg/d) Fludarabine (6 × 30 mg/m^2^) Cyclophosphamide (2 × 20 mg/kg/d)	post-transplant period was uneventful, and 6 months after SCT cyclosporine was stopped because of decreasing donor chimerism.	[53]
6	4 years	Upper respiratory infections; skin and joint granulomas; very low naïve T cells; absence of IgA; low IgG2 and IgG4, alpha fetoprotein (AFP) level of 52 ng/mL (normal range <7); Total serum IgG and IgM normal	2012	HLA-identical sibling	Fludarabine (5 × 30 mg/m^2^/d), Cyclophosphamide (4 × 20 mg/kg/d) ATG-Fresenius (20 mg/kg)	AlloHSCT corrected the T-cell lymphopenia by expansion of naïve and memory CD4+ T-cells, CD19+ cells, and CD8+ T-cells; gain in height and weight; complete remission of skin and joint granulomas; milder progression of ataxic symptoms.	[55]

Notes: N/S: not specified; BMT: bone marrow transplantation; ATG-Fresenius: anti-thymocyte globulin; Allo-HSCT: allogeneic hematopoietic stem cell transplantation; HLA: human leukocyte antigen; GvHD: graft-versus-host disease; Busilvex: busulfan, Pierre Fabre, Freiburg, Germany; I.V.: intravenous.

**Table 2:** Summary of bone marrow transplantation in A-T children.

Case	Conditioning Protocol Prior to Allo-HSCT	Outcome
1	N/S	Death due to hepatic failure
2	Modified GEFA02	Leukemia remission
3	GEFA02	CD8+ and CD4+ T cells normalized with no GvHD
4	GEFA03	Poor immunologic recovery and low donor chimerism
5	Modified GEFA03	Stable CD4+ and CD8+ T cell counts
6	N/S	Renewal in peripheral blood cells and ATM protein expression

Notes: Allo-HSCT: allogenic hematopoietic stem cell transplantation; N/S: not specified; GEFA: German Fanconi anemia; GvHD: graft-versus-host disease.

## Abbreviations

A-T	Ataxia-Telangiectasia;
ATM	Ataxia-Telangiectasia mutated;
BMT	bone marrow transplantation;
DSBs	double strand breaks;
MRN	MRE11/RAD50/NBN;
V(D)J	variable (V), diversity (D) and joining (J);
AFP	alphafetoprotein;
SOD	superoxide dismutase;
ROS	reactive oxygen species;
IR	irradiation;
IGF-1	insulin-like growth factor 1;
SMRT	small molecule read-through;
ASO	antisense oligonucleotides;
CRISPR/Cas9	clustered regularly interspaced short palindromic repeats/CRISPR-associated protein-9;
iPS cells	induced pluripotent stem cells;
HSV-1	Herpes simplex virus type 1 AAV: adeno-associated virus;
HSCs	hematopoietic stem cells;
BMDCs	bone marrow-derived cells;
GFP	green fluorescent protein;
Allo-HSCT	allogenic hematopoietic stem cell transplantation;
GvHD	graft-versus-host disease;
EBV	Epstein−Barr virus;
HLA	human leukocyte antigen;
RIC	reduced intensity conditioning.
